# Bisphosphonates and Their Connection to Dental Procedures: Exploring Bisphosphonate-Related Osteonecrosis of the Jaws

**DOI:** 10.3390/cancers15225366

**Published:** 2023-11-10

**Authors:** Emily Sunny Lee, Meng-Chen Tsai, Jing-Xuan Lee, Chuki Wong, You-Ning Cheng, An-Chi Liu, You-Fang Liang, Chih-Yuan Fang, Chia-Yu Wu, I-Ta Lee

**Affiliations:** 1 School of Dentistry, College of Oral Medicine, Taipei Medical University, Taipei 11031, Taiwan; b202111074@tmu.edu.tw (E.S.L.); b202111039@tmu.edu.tw (M.-C.T.); b202111006@tmu.edu.tw (J.-X.L.); b202111077@tmu.edu.tw (C.W.); b202111020@tmu.edu.tw (Y.-N.C.); b202111029@tmu.edu.tw (A.-C.L.); b202111009@tmu.edu.tw (Y.-F.L.); d204106002@tmu.edu.tw (C.-Y.F.); 2 Division of Oral and Maxillofacial Surgery, Department of Dentistry, Wan Fang Hospital, Taipei Medical University, Taipei 11696, Taiwan; 3 Division of Oral and Maxillofacial Surgery, Department of Dentistry, Taipei Medical University Hospital, Taipei 11031, Taiwan; 4 School of Dental Technology, College of Oral Medicine, Taipei Medical University, Taipei 11031, Taiwan

**Keywords:** bisphosphonates, cancer, bisphosphonate-related osteonecrosis of the jaws, inflammation, dentistry

## Abstract

**Simple Summary:**

We delve into the intricate relationship between bisphosphonates, commonly used for treating osteoporosis and tumors, and their potential side effect: bisphosphonate-related osteonecrosis of the jaws (BRONJ) after dental procedures. We explore how bisphosphonates affect bone density and resorption, emphasizing their impact on dental treatments. Dental patients on bisphosphonates face a heightened risk of BRONJ, especially post-invasive dental procedures. Although topical applications enhance dental implant success and aid in periodontal treatment, systemic administration significantly raises BRONJ risk. Preventive measures involve maintaining oral health and considering alternative treatments. This review underscores the need for further research to refine protocols and ensure the safety of dental procedures for bisphosphonate-treated patients.

**Abstract:**

Bisphosphonates are widely used to treat osteoporosis and malignant tumors due to their effectiveness in increasing bone density and inhibiting bone resorption. However, their association with bisphosphonate-related osteonecrosis of the jaws (BRONJ) following invasive dental procedures poses a significant challenge. This review explores the functions, mechanisms, and side effects of bisphosphonates, emphasizing their impact on dental procedures. Dental patients receiving bisphosphonate treatment are at higher risk of BRONJ, necessitating dentists’ awareness of these risks. Topical bisphosphonate applications enhance dental implant success, by promoting osseointegration and preventing osteoclast apoptosis, and is effective in periodontal treatment. Yet, systemic administration (intravenous or intraoral) significantly increases the risk of BRONJ following dental procedures, particularly in inflamed conditions. Prevention and management of BRONJ involve maintaining oral health, considering alternative treatments, and careful pre-operative and post-operative follow-ups. Future research could focus on finding bisphosphonate alternatives with fewer side effects or developing combinations that reduce BRONJ risk. This review underscores the need for further exploration of bisphosphonates and their implications in dental procedures.

## 1. Introduction

**Figure 1:**
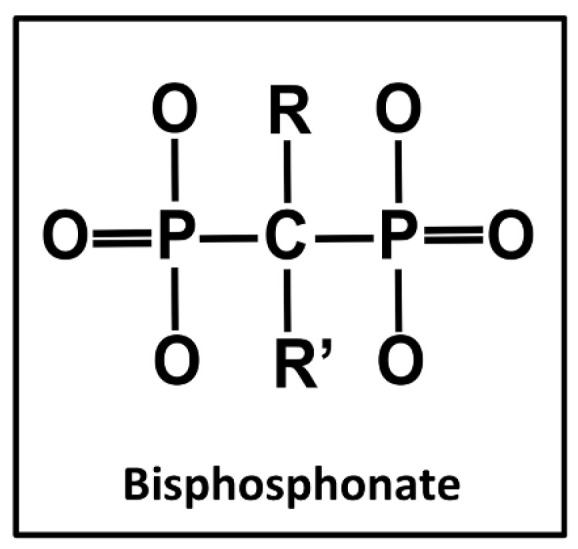
Chemical structure of bisphosphonates. The “R” and “R’” refers to long carbon chains that differ depending on the type of bisphosphonate.

Bisphosphonates, a class of medications frequently employed to treat bone-related diseases, function by enhancing bone formation, improving bone density, and inhibiting osteoclast activity and bone resorption [1,2,3]. Structurally, bisphosphonates consist of two phosphonate groups linked by a carbon atom, rendering them resistant to degradation in acidic environments or by pyrophosphatases [4,5]. Figure 1 illustrates the general chemical structure of bisphosphonates, with the attached carbon chains (denoted as R and R’) varying depending on the specific type of bisphosphonate [6]. These medications can be administered orally or intravenously. Intravenous bisphosphonates, being more potent, are utilized for conditions like Paget’s disease, bone pain, and malignant tumors, while oral bisphosphonates are commonly used to treat osteoporosis, particularly in the elderly [1,7]. The most common oral bisphosphonates include alendronate and risedronate, whereas pamidronate and zoledronate are usually administered intravenously; some bisphosphonates are given both orally and intravenously, depending on the treatment and patient’s physical conditions [8]. Despite their efficacy, bisphosphonates are associated with upper gastrointestinal side effects, such as dysphagia, nausea, vomiting, and dyspepsia [9]. Their strong affinity for bone hydroxyapatites prevents bone degeneration and calcification, impacting bone development [10]. By impeding osteoclast recruitment, reducing activity, and inducing osteoclast apoptosis, bisphosphonates effectively decrease osteoclast activity and numbers [1,11,12]. Osteoporosis, a progressive skeletal disorder resulting in bone tissue loss and increased fragility, necessitates bisphosphonate treatment to strengthen bones and prevent fractures [2,13]. However, the use of bisphosphonates is associated with a severe side effect, especially relevant in dentistry: bisphosphonate-related osteonecrosis of the jaws (BRONJ) [8,14]. BRONJ typically occurs after invasive dental procedures, such as tooth extraction, oral trauma, or infections, particularly in the maxilla and mandible, where bisphosphonates tend to accumulate [8,12]. It manifests as visible necrotic bone in the jaws lasting more than eight weeks in individuals undergoing bisphosphonate therapy; the untreated bone can be seen and identified radiographically [12,15,16]. Even minor injuries can lead to impaired jawbone tissue repair in this state [16]. Given the widespread use of bisphosphonates in cancer treatment and managing osteoporosis—especially prevalent among the elderly—dental procedures pose significant challenges. When major dental procedures are necessary for patients receiving bisphosphonate therapy, careful management is crucial to ensure the success of treatment, as well as the health and safety of the patient. In this paper, we aim to address the interconnectedness of bisphosphonate, BRONJ, and their relations to dental procedures, namely implants, extractions, endodontics, periodontal treatment, and prosthodontics. We narrowed the scope of our search to only include statistics from studies published after 2010, in order to ensure the recency of the findings. Additionally, we suggested clinical guidelines and procedures to better mitigate the risks of BRONJ in bisphosphonate patients.

## 2. BRONJ: Mechanisms, Clinical Stages, and Effective Management Strategies

### 2.1. Mechanisms

**Figure 2:**
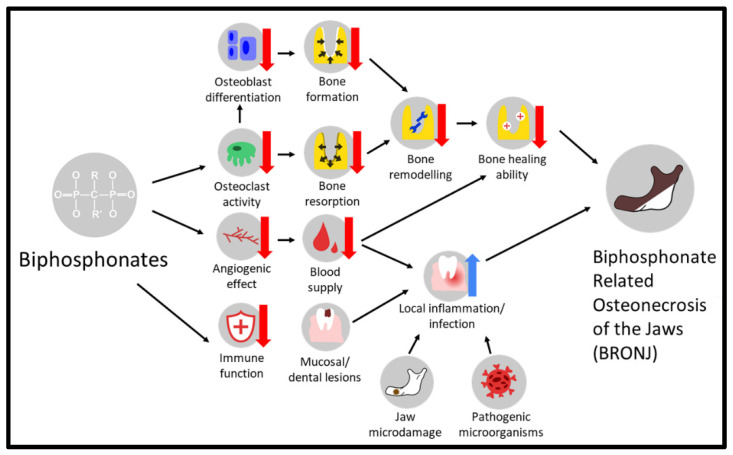
An overview of potential factors contributing to the development of BRONJ.

The intricate mechanisms underlying BRONJ are not yet fully understood. However, its development is believed to result from various contributing factors. Figure 2 visually outlines the intricate interconnections among these factors, shedding light on the complex nature of BRONJ.

#### 2.1.1. Bone Remodeling Inhibition

One prevailing theory revolves around the inhibition of bone remodeling. Bisphosphonates exhibit a strong affinity for hydroxyapatite crystals, the primary constituents of bone mineral [17,18]. This affinity results in the induction of apoptosis and suppression of the activity of osteoclasts, the cells responsible for bone resorption [18,19,20]. By impeding osteoclast function, bisphosphonates disrupt the natural process of bone remodeling, causing delays and impairments in bone healing, particularly following invasive dental surgeries [21]. Furthermore, bisphosphonates indirectly hinder the differentiation of osteoblasts due to the absence of cytokines released by osteoclasts. This disruption further compromises the bone’s ability to heal and escalates the risk of osteonecrosis [18]. Notably, an analysis of human bone specimens from patients undergoing bisphosphonate treatment reveals an increased presence of nonfunctional osteoclasts surrounding necrotic bones, reinforcing the hypothesis of bone remodeling inhibition as a significant factor in the development of BRONJ [22,23]. This intricate interplay between bisphosphonates, osteoclasts, and osteoblasts underscores the complexity of BRONJ’s pathogenesis. The inhibition of bone remodeling disrupts the delicate balance between bone resorption and formation, playing a pivotal role in the onset and progression of this condition. The combination of apoptosis induction, osteoclast suppression, and impaired osteoblast differentiation creates a microenvironment conducive to osteonecrosis, highlighting the multifaceted nature of BRONJ’s development [18,19,20,21,22,23].

#### 2.1.2. Angiogenesis Inhibition

Bisphosphonates are known to interfere with angiogenesis, inhibiting the formation of new blood vessels within the jawbone [24]. This impaired blood supply can impede proper bone healing, making the tissue more susceptible to infections and necrosis [23]. Studies conducted on animals have demonstrated a reduction in arterial area, venous area, and overall vascularity in periodontal tissues during the onset of BRONJ [25,26]. Remarkably, research indicates a higher incidence of BRONJ in individuals simultaneously using anti-resorptive agents (such as bisphosphonates) and anti-angiogenic agents [27,28]. The cumulative evidence highlights the significant impact of bisphosphonates on diminishing vascularity in jawbones. This decrease in blood vessel formation compromises the natural healing capacity of bones, elevating the risk of complications such as infections and necrosis. This intricate relationship between the inhibition of angiogenesis and the development of BRONJ underscores the necessity for a comprehensive understanding of vascular dynamics in bone tissue, particularly concerning bisphosphonate treatments [24,25,26,27,28]. It emphasizes the importance of ongoing research to delve deeper into these interactions, enabling healthcare professionals to make informed decisions and implement targeted strategies when managing patients undergoing bisphosphonate therapy. This knowledge is paramount in enhancing patient outcomes and ensuring the safety and efficacy of treatments in the realm of bone-related conditions and cancer therapies.

#### 2.1.3. Inflammation or Infection

Another hypothesis centers on the immunosuppressive effects of bisphosphonates on the body’s immune system [29]. These effects have the potential to compromise the jawbone tissues’ ability to defend against infections. Mucosal or dental lesions associated with bisphosphonates can trigger local inflammation, leading to infections that spread to the bone and exacerbate osteonecrosis [30]. Several factors contribute to the specificity of bisphosphonate-related osteonecrosis in the jaw. Firstly, the mandibular and maxilla bones are thinly covered by a mucoperiosteal layer, offering limited protection against oral pathogens [30]. Furthermore, the jawbone is prone to repetitive microtraumas due to occlusion and chewing activities. This ongoing bone remodeling process facilitates a higher accumulation of bisphosphonates, magnifying their impact [30]. Specifically, within the jaw, the alveolar crest undergoes a faster rate of remodeling compared to other bones, resulting in the accumulation of one of the highest concentrations of bisphosphonates [17,30]. This complex interplay between immunosuppression, recurrent microtraumas, and the unique anatomical characteristics of the jawbone underscores the specific vulnerability of the jaw to bisphosphonate-related osteonecrosis. Understanding these intricate factors is essential for developing targeted and effective preventive strategies and treatment approaches [29,30]. Continued research in this area is vital to advancing our knowledge and enhancing the care and safety of individuals undergoing bisphosphonate therapy.

### 2.2. Clinical Manifestations and Staging

In clinical settings, BRONJ exhibits a range of symptoms, the severity of which can vary. These symptoms commonly include jaw pain, mobility of teeth, exposed jawbone, soft tissue damage or ulceration, numbness of the lower lip (hypoesthesia), non-healing extraction sockets, and swelling or inflammation in the jaw and gum area [31,32]. The progression of BRONJ is categorized into distinct stages. Table 1 provides a concise summary of the key points of each stage.

According to the American Association of Oral and Maxillofacial Surgeons’ (AAOMS) Position Paper, four stages can be observed:

Stage 0: This stage is characterized by nonspecific symptoms such as pain and swelling, lacking clinical evidence of exposed bone or necrotic tissue [32,33].

Stage 1: Patients in this stage are asymptomatic but exhibit exposed and necrotic bone within the mouth, persisting for more than eight weeks without signs of infection [32,33].

Stage 2: In this stage, there is exposed and necrotic bone (fistulae) that can be probed down to the bone. This condition is associated with pain and infection, indicating a more advanced level of tissue involvement [32,33].

Stage 3: The most severe stage involves extensive exposed and necrotic bone (fistulae) that can be probed down to the bone. It is associated with active infection and additional complications such as pathological fractures of the jaw, representing a critical and challenging clinical scenario [32,33].

Alternatively, a different approach to BRONJ staging has been proposed by the Italian Society of Maxillofacial Surgery and the Italian Society of Oral Pathology and Medicine, jointly known as the SICMF-SIPMO. In particular, the criteria proposed by SICMF-SIPMO emphasizes the importance of radiological symptoms that can be observed via CT scans, whereas AAOMS considers only clinical symptoms [34]. SICMF-SIPMO does not have a stage 0, and classifies stages in the following way:

Stage 1: Focal, patients exhibit at least one minor clinical symptom; CT scans showing bone condensation limited to only the alveolar process [34].

Stage 2: Diffused, patients exhibit at least one minor clinical symptom; CT scans showing bone condensation spreading to the basal bone area [34].

Stage 3: Severe, patients exhibit one or more major clinical symptoms; CT scans showing large scale damage, such as fractures or osteolysis, of the surrounding facial bones, (e.g., mandible, maxilla, zygomatic or hard palate) [34].

Regardless of the differences in the staging criteria, these progressive stages highlight the varying degrees of tissue involvement and clinical complexity in BRONJ cases. Understanding these stages is essential for accurate diagnosis, appropriate management, and tailored treatment planning for affected individuals [32,33].

### 2.3. Risk Factors

As previously stated, bisphosphonates serve as the primary trigger for BRONJ. Nevertheless, the development of BRONJ is influenced by various additional risk factors.

#### 2.3.1. Accumulated Dosage (Prolonged Treatment Duration)

The accumulation of medication over time often occurs during extended treatment, a situation more frequently encountered in cancer patients [35]. Extensive research on individuals undergoing cancer treatment has unequivocally established a direct link between the prevalence of BRONJ and the duration of exposure to bisphosphonate drugs. This research underscores the significantly heightened risk associated with prolonged treatment periods [35]. Consequently, it is imperative to emphasize the critical importance of monitoring the duration of bisphosphonate therapy, especially in cancer patients, in order to mitigate the potential occurrence of BRONJ. This correlation emphasizes the need for vigilant oversight and careful management of patients undergoing extended bisphosphonate treatments. It calls for a collaborative effort between oncologists and dental professionals, working closely together to ensure a nuanced and balanced approach. Such an approach must not only take into account the therapeutic benefits these treatments offer, but also carefully weigh them against the potential risks associated with their prolonged use [35]. By doing so, healthcare providers can optimize patient outcomes while minimizing the adverse effects, thereby enhancing the overall quality of care for individuals requiring extended bisphosphonate therapy.

#### 2.3.2. Dental Surgical Procedures

The association between dental extraction and osteonecrosis of the jaw has been firmly established. Patients with dentures face a heightened risk in this regard [36]. Clinical studies revealed a significant increase in the risk of developing BRONJ following dental extraction, with figures ranging from 33 to 333 times higher compared to non-extraction cases [37]. Moreover, tooth extraction was found to trigger osteonecrosis in 38% of patients [38]. Several studies have emphasized the role of concurrent dental diseases or dentoalveolar procedures in elevating the risk of BRONJ [38,39]. It is worth noting that whether BRONJ manifests after invasive oral surgical procedures in patients on bisphosphonates often hinges on the expertise and technique employed by the dentist [37,39,40]. This body of evidence highlights the critical need for meticulous dental care and skilled dental practitioners when treating patients undergoing bisphosphonate therapy, especially those requiring dental extractions or similar interventions [36,37,38,39,40]. Details about the interconnectedness of BRONJ and specific dental procedures will be addressed in detail in the next section.

#### 2.3.3. Malignancy

Cancer chemotherapy medications and the presence of malignancies significantly elevate the risk of jaw osteonecrosis [41]. Cancer cells often metastasize to the bones, necessitating the use of bisphosphonates to alleviate symptoms and impede further damage. Paradoxically, the essential administration of bisphosphonates to manage bone complications in cancer patients simultaneously heightens the risk of developing BRONJ [42,43,44]. This double-edged relationship between cancer treatments, bisphosphonate usage, and the increased susceptibility to BRONJ underscores the intricate challenges faced in managing cancer-related bone issues. Balancing the therapeutic benefits of bisphosphonates with the potential complications, especially in cancer patients, necessitates careful consideration and close monitoring to mitigate the risk of BRONJ effectively [41,42,43,44].

### 2.4. Prevention and Management

Given the high occurrence rate of jaw osteonecrosis in patients with malignant diseases (92%), it is imperative to establish effective prevention and management strategies [35,37]. A structured approach can be taken to address patients at different stages of bisphosphonate treatment. For individuals about to commence intravenous bisphosphonate therapy, the primary objective is to minimize the risk of BRONJ by optimizing dental health [45]. Ideally, bisphosphonate therapy initiation should be delayed until dental health is thoroughly optimized, a decision that requires collaboration among the treating physician, dentist, and specialists. During this stage, the focus should be retaining restorable teeth and completing necessary surgeries 4 to 6 weeks before initiating bisphosphonate treatment, allowing adequate time for osseous healing; patients should actively cooperate with regular evaluations and promptly report any symptoms potentially related to BRONJ [33,45]. The next stage involves patients already receiving bisphosphonates, but without evidence of osteonecrosis [33,45]. Here, maintaining excellent oral hygiene remains crucial, with a focus on preserving teeth whenever possible. If tooth extraction becomes necessary, retaining the tooth root is recommended, to minimize trauma to the bone and oral tissues. Additionally, patients exposed to bisphosphonates like zoledronic acid and pamidronate should avoid dental implant procedures, minimizing the risk of complications. The final stage pertains to patients diagnosed with established BRONJ [33,45]. In these cases, panoramic and tomographic imaging are essential for accurate assessment. However, due to the unpredictable response of these cases to bisphosphonates, surgical treatment is challenging and should be delayed. During this stage, trimming soft tissues helps minimize exposure and promotes healing. The primary objectives at this stage include pain elimination and infection control, highlighting the critical importance of comprehensive and personalized management strategies [37,46,47]. This systematic approach is vital for enhancing the quality of care and outcomes for patients dealing with BRONJ.

## 3. Bisphosphonates’ Impact on Dental Procedures

### 3.1. Dental Implants

Implantology, a dental procedure involving the placement of dental implants in the maxillary or mandibular bone, is vital for restoring missing teeth, supporting chewing function, and preserving aesthetics [48]. Key to the success of dental implants is the process of osseointegration, the formation of integration between bone and titanium implants, a process influenced by factors like bone characteristics, tobacco use, and the patient’s overall health [49,50]. Bisphosphonates, commonly used for medical treatments, can be administered systematically or topically, the latter involving localized application, such as coating the surface of dental implants [51]. Studies have shown positive outcomes when bisphosphonates are coated on implants, enhancing osseointegration and delaying osteoclast apoptosis, ultimately extending the preservation time of marginal bone around implants [51,52,53]. These findings indicate the potential benefits of topical bisphosphonate applications, presenting a promising avenue for clinical consideration. However, the impact of BRONJ on dental implants depends on the method of administration. Research indicates that patients using intraoral bisphosphonates for less than five years pose a low risk for BRONJ following dental implantation, especially when operative care is meticulously observed [54]. In contrast, intravenous bisphosphonate therapy carries a higher risk, making dental implant placement a high-risk situation for these patients [55]. To ensure implant safety, pre-operation measures such as drug withdrawal evaluation, antibiotic usage, and follow-up protocols are essential [3,56]. Additionally, combining treatments, such as using plasma rich in platelet (PRP), platelet-rich fibrin (PRF), and plasma rich in growth factors (PRGF), enhances wound closure and aids in the implant healing process [57,58,59]. In conclusion, the method and duration of bisphosphonate administration significantly influences dental implant outcomes and the risk of developing BRONJ. Continued research and in-depth studies are imperative to gain a comprehensive understanding of bisphosphonate effects on implantology. Dentists must remain vigilant, thoroughly evaluating patient conditions, and maintaining rigorous follow-up protocols post-implantation, especially for patients undergoing bisphosphonate treatments, to mitigate the risk of BRONJ and ensure the success of dental implant procedures.

### 3.2. Extraction

Based on research and statistical findings, BRONJ typically manifests following tooth extraction [60]. Most studies suggest that the development of BRONJ is influenced by various contributing factors. One study revealed that patients currently undergoing intravenous bisphosphonate therapy did not exhibit a higher risk for BRONJ compared to patients who had completed or temporarily paused their intravenous bisphosphonate therapy [61]. However, the risk of BRONJ significantly increases when tooth extraction involves an osteotomy, particularly a mandible osteotomy [61]. Several investigations propose that common infectious diseases are also contributing factors to the development of BRONJ [62,63]. For instance, preexisting pathological inflammatory conditions like baseline osteomyelitis and periapical periodontitis are risk factors that may induce the development of BRONJ following tooth extraction [64,65]. Otto et al. concluded that tooth extraction itself might not be the primary cause of BRONJ but rather the complications, such as infections associated with the procedure, contribute to its development [65]. On the contrary, Song et al. found that "tooth extraction alone" could lead to BRONJ, while inflammation induced by pulp exposure worsened BRONJ by causing more bone necrosis [65]. BRONJ did not develop when inflammation was present without a tooth extraction procedure [65]. In this case, tooth extraction appears to act as a trigger, but other factors can influence the severity of BRONJ. Similarly, tooth extraction was reported to be the leading cause of BRONJ among patients with bisphosphonate therapy, with a relative risk “5.3–53 times higher” than bisphosphonate patients who do not undergo tooth extraction [66]. Since extractions appear to be a high-risk trigger for the development of BRONJ in bisphosphonate patients, it is recommended to avoid extractions, if possible, but only under conditions that allow for the lesion or inflammation to be resolved with alternative treatments [66].

On the other hand, although patients administered bisphosphonates are currently classified as high-risk, some believe tooth extraction can be performed safely following established guidelines [62,63]. An experimental study indicated that the application of bFGF, a growth factor, can promote healing in tooth sockets after extraction and prevent the development of BRONJ. Researchers suggested that bFGF might counteract the interference of bisphosphonates with bone healing. Specifically, bFGF prevents the inhibition of healing, thereby reducing the risk of BRONJ after tooth extraction [60]. Treatment with PRGF, another growth factor, can restore the osteoblast/osteoclast homeostatic cycles, counteracting and minimizing the effects of bisphosphonates on osteoclasts, thus reducing the risk of BRONJ development in patients [67]. In another case, a patient with BRONJ was treated by removing infected tissue and suspending bisphosphonate usage, with no signs of recurrence after a year [68]. While the precise role of tooth extraction in the development of BRONJ may still require further investigation, it is evident that maintaining good oral health is beneficial in preventing severe BRONJ, either by decreasing its severity or by reducing the risk of its development.

### 3.3. Endodontics

Due to the high risk of developing BRONJ following tooth extraction in bisphosphonate patients, endodontics is commonly seen as an alternative treatment method. Endodontics could preserve the tooth, preventing the need for extraction and significantly decreasing the risk of apical periodontitis, both of which majorly contribute to the development of BRONJ [69]. However, endodontics itself could also contribute to BRONJ. During root canal shaping and cleaning, the inner pulp and dentin of the tooth is directly exposed to the oral environment and the microorganisms within [69]. Moreover, soft tissue damage can occur while cleaning the root or when applying a rubber dam, also increasing the risk of BRONJ [70].

Exploring this relationship inversely, bisphosphonates impact the outcome of endodontic treatments. After root canal treatment, periapical lesions heal via a bone remodeling process, but this process would be inhibited by bisphosphonates [70]. The lack of bone modelling delays the healing process, and, if coupled with microleakage or incomplete seals, could increase the risk inflammation, thus also increasing the risk of BRONJ developing [70,71]. 

That being said, it appears endodontic procedures performed on bisphosphonate patients generally have high success rates. Studies have found a high tooth survival rates of endodontic teeth of bisphosphonate patients of around 70%, with no significant difference compared to the control group; extractions were only deemed necessary in the case of tooth fractures and periodontal inflammation [72,73]. Oral bisphosphonates were not found to pose a significant risk to the successful healing following endodontic treatment [74]. On the other hand, IV bisphosphonates may interfere with the healing of root canal procedures: Dereci et al. found endodontic procedures to be more successful in patients receiving IV bisphosphonates less than a year compared to those that have been for a longer period; however, this study has a small sample size, and the generalizability of the results may be limited [75]. Although endodontic procedures in bisphosphonate patients may not be without risks, it appears that it might be a suitable alternative for tooth extraction in bisphosphonate patients, if conditions permit. 

### 3.4. Periodontal Treatment

Periodontitis is the inflammation of the gingiva caused by bacterial flora, which could lead to the destructive of gingival tissues, or tooth loss, in more severe cases [76]. It can be caused by both local and systemic factors, and is easily influenced by factors such as immunity, oral hygiene habits, and many more [77]. Periodontitis is traditionally treated via a procedure known as scaling and root planning (SRP), which physically removes the pathogenic microorganisms causing the inflammation [76]. However, many studies have found adjunctive bisphosphonate therapy to be effective in the treating periodontitis, due to the bisphosphonates’ ability to inhibit proinflammatory cytokines and the resorption of alveolar bone, providing a different approach to periodontal treatment [78,79,80,81]. Bisphosphonates have been administered intravenously or applied locally, in gel-form (often using alendronate specifically) for these purposes [82,83]. According to several review articles, a vast number of studies with locally applied bisphosphonates (applied after traditional SRP) showed significant improvements to patients’ periodontal conditions, resulting in decreased probing pocket depth [76,78]. Similar results were found for bisphosphonates administered systemically, whether orally or intravenously, with oral bisphosphonates showing greater improvement compared to IVs [76]. Although both systemic and local bisphosphonates demonstrate effectiveness, locally applied bisphosphonates are preferred due to having fewer side effects [78]. Based on these results, it seems quite promising to utilize bisphosphonates in conjunction with SRP for periodontal treatment. There has not yet been a comparison of effectiveness between locally and systemically applied bisphosphonates for periodontal treatment, which is a potential area for further research. 

However, periodontitis itself is closely correlated with BRONJ. According to Thumbigere-Math et al., a high percentage of patients with BRONJ had periodontal disease, ranging from 41–84% [84]. BRONJ patients were found to have less alveolar bone support and fewer teeth remaining, which are both indirect measures of periodontal health [84]. Microorganisms causing periodontitis have also been found in BRONJ lesions from multiple studies [85,86]. The American Association of Oral and Maxillofacial Surgeons’ Position Paper (2022 Update) also cites “pre-existing inflammatory dental disease such as periodontal disease” as a risk factor for BRONJ [23]. 

It is quite interesting to consider bisphosphonates being related to periodontal disease and BRONJ from two perspectives. On one hand, bisphosphonates are the solution; they can help improve periodontal disease through decreasing inflammatory cytokines and inhibiting bone resorption [78,79,80,81]. But on the other hand, bisphosphonates coupled with periodontal diseases poses the risk of BRONJ developing. A recent study, conducted in 2021, found results indicating that the use of systemic bisphosphonates along with periodontal disease leads to the development of BRONJ in rats [21]. They proposed a possible mechanism: the inflammation from periodontal disease coupled with the decreased osteoclastic activity due to bisphosphonates exposes the alveolar bone to a high concentration of inflammatory cytokines and microorganisms, both of which increases the risk of BRONJ developing [21]. 

Taken together, this raises the following question: is it worth it to introduce bisphosphonates—for the sake of improving periodontal conditions—into a patient who has no prior history of using bisphosphonates, thus presenting the possibility of developing BRONJ? Without dental bisphosphonate treatment, these patients have no risk of BRONJ, even with their present periodontitis conditions. However, periodontitis is a problem that significantly affects the oral and general health of the patient; it should be addressed regardless of its connection to BRONJ. Based on the data, bisphosphonates seem to be quite effective to use in conjunction with SRP, but some researchers have suggested against using it for reasons relating to BRONJ [78]. The decision of whether or not bisphosphonates should be applied in the treatment of periodontitis should be a mutual agreement between doctors and patients, and should be made after careful consideration of the patient’s health conditions and other relevant factors.

### 3.5. Prosthodontics

The relationship between bisphosphonates and prosthodontics, whether fixed or removable, has not been studied in depth, and a consensus cannot be reached regarding its role in the development of BRONJ. Some studies identified the use of dentures as a risk factor for BRONJ developing, attributing the reason to denture trauma [87,88,89]. Contrarily, other researchers stated that prosthodontic management does not pose a high risk, especially when compared with aforementioned invasive oral surgical procedures, namely extractions and implants, even suggesting that patients who have developed BRONJ can still undergo prosthodontic rehabilitation for aesthetic and functional purposes [90]. Nevertheless, it is beneficial to minimize the pressure of the dental prosthesis on the mucosa to minimize the risk of BRONJ, or to avoid worsening the condition [87,90]. Removable dentures were identified to be more likely to cause mucosa trauma, compared to fixed dentures [90]. A heat-polymerized resilient denture liner as a denture base material has been suggested to prevent localized stresses and distributing the load more evenly over the whole base, potentially providing a preventative measure for patients requiring prosthodontics [87]. It is critical that patients with dentures attend dentist appointments regularly. Any discomfort should be promptly reported so poorly fitting dentures can be adjusted or replaced to minimize soft tissue trauma [89]. Ali et al. recommended closely monitoring the denture-bearing tissues and prosthesis at 2–3-month intervals [90].

## 4. Discussion

In this review, we have explored the impact of bisphosphonates on dental procedures, focusing on dental implants, extractions, endodontics, periodontal treatment, and prosthodontics. The findings presented here provide valuable insights into the complexities surrounding bisphosphonate administration and its effects on oral health.

### 4.1. Administration Routes and Effect on Dental Procedures

Dental procedures are affected by bisphosphonates differently, but general trends can be suggested based on current research. In terms of systemic bisphosphonates, it has been found that BRONJ has higher incidence rates in bisphosphonate patients if they were administered intravenously, compared to oral bisphosphonates, regardless of the dental procedure performed [54,55,66]. We cannot be conclusive that the differences are caused by the route of administration alone; other factors, such as differing health conditions of patients who require IV or oral bisphosphonates could also play a role. However, a recent study has found that IV administration of bisphosphonates seem to have a greater effect on osteoclastic activity—compared to oral bisphosphonates at the same dosage—that seems to “aggravate” the conditions of BRONJ [91]. 

As opposed to systemic bisphosphonates, locally applied bisphosphonates seem to be an overall positive addition to dental procedures. Studies show that it positively improves implant osseointegration and periodontal treatment [51,52,53,78,79,81]. There has been no record of BRONJ occurring after applying bisphosphonates locally, and the general consensus seems to be that locally applied bisphosphonates pose no serious side effects to the patient [52,78]. Current literature does not seem to explain the mechanisms or reasons behind this phenomenon; this gap in knowledge is an important area in need of further research. 

### 4.2. Interconnectedness of Risk Factors of BRONJ

Additionally, although tooth extractions are commonly viewed as a major contributing factor to BRONJ, some researchers believe the involvement of tooth extractions is overstated. For one, Kishimoto et al. proposes that most tooth extraction are necessary because of periodontal inflammation [92]. Hence, it may appear that tooth extraction is the leading cause of BRONJ, when other factors, such as periodontal disease, could also be contributing to its development. It seems possible that extractions are not the sole reason for BRONJ, and BRONJ sometimes develop even in the absence of extractions; however, the act of extracting seems to be a major trigger that greatly increases the risk of BRONJ developing soon after [65]. It is also important to note that the process of tooth extraction was found to increase inflammatory cytokine levels—a major risk factor of BRONJ—which would not be present in the absence of extractions; thus, perhaps the act of extraction does play a significant role in BRONJ [93]. Regardless of whether it is extractions themselves or the combination of extractions and periodontal inflammation increasing the incidence rate of BRONJ, it is crucial not to delay extractions due to fear of BRONJ developing. The untreated inflammation poses a serious risk for BRONJ and may lead to other oral health conditions. If the patient’s circumstances allow for it, endodontic treatment could be used as an alternative to extraction, decreasing the risk of microdamage or trauma that could trigger BRONJ.

### 4.3. Clinical Guidelines for Minimizing Risks

Due to the prevalence of bisphosphonates used in medical treatment, it seems necessary to propose guidelines for preventative measures and clinical recommendations to help minimize the risk of BRONJ. A summary of pre-procedural and post-procedural guidelines for both patients and doctors can be found in Table 2. Generally agreed upon guidelines include: identifying and treating infection or potential infection sites, performing high-risk operations (e.g., tooth extractions, dental implants) prior to starting bisphosphonate therapy, maintaining good oral and general health, using antimicrobial mouthwash (e.g., chlorhexidine) prior and following procedures, thoroughly sanitizing equipment, and minimizing trauma during dental procedures [24,70]. These preventative measures were found to significantly reduce risk of BRONJ developing [94,95].

The need or effectiveness for a drug holiday is considered controversial. Some researchers support it for the purposes or minimizing BRONJ risk, while others believe it interferes with the original intention of the bisphosphonate treatment in addition to increasing the risk of bone fractures during the dental procedure, especially in the case of osteoporosis patients [23]. Additionally, antibiotics have been suggested to decrease local inflammation and reduce inflammatory cytokines prior and following major dental procedures [93,96,97]. While antibiotics might be useful, there is debate regarding their necessity, addressing concerns surrounding antimicrobial resistance [98]. The overuse of antibiotics in dentistry is a phenomenon not limited to BRONJ, and bisphosphonate patients are at a significantly higher risk of developing devastating side effects; thus, antibiotic use does not seem unjustified in this case, but ultimately, it is the doctor’s decision. 

The usage of anesthetic agents without vasoconstricting effects has also been suggested [70,99]. As bisphosphonates already have angiogenic effects that increase risk of BRONJ, some researchers want to prevent further reducing blood flow to the operated area [70,99]. However, this comes with greater risks, such as extreme blood loss for the patient, and thus, is not generally incorporated in dental procedures. 

## 5. Conclusions

In conclusion, while bisphosphonates offer significant benefits in various medical and dental treatments, their impact on oral health and other dental procedures necessitates careful consideration. Systemic administration of bisphosphonates generally poses a risk of BRONJ developing following major dental procedures, such as implants and extractions; thus, endodontics could be a lower-risk alternative for extractions, if conditions permit. Local applications of bisphosphonate greatly increase the success rates of periodontal treatment and implant osseointegration. Continued research in this field is essential to refine protocols, ensuring the safety and success of dental procedures for patients undergoing bisphosphonate treatments.

## Figures and Tables

**Table 1:** A summary table of the symptoms and clinical stages of BRONJ, according to AAOMS and SICMF-SIPMO.

	AAOMS		SICMF-SIPMO
Stage	Symptoms	Clinical Evidence	Radiological Symptoms (CT Results)
Stage 0	Nonspecific; pain and swelling	Lacking (difficult to diagnose)	N/A
Stage 1	Asymptomatic	Exposed and necrotic bone, but no infection, for at least 8 weeks	Focal: Bone condensation limited to only the alveolar process
Stage 2	Pain and infection	Exposed and necrotic bone (with infection)	Diffused: Bone condensation spreading to the basal bone area
Stage 3	Active infection, with complications, e.g., pathological fractures of the jaw	Extensive exposed and necrotic bone (with active infection)	Severe: Large scale damage, such as fractures or osteolysis, of facial bones

**Table 2:** A summary of clinical guidelines for minimizing risk of BRONJ.

Role of Patient	Role of Doctor	Notes
Pre-procedure		
Maintaining good oral health (e.g., brushing teeth, flossing, etc.) Maintaining good general health (e.g., smoking cessation, controlling diabetes)	Inform patient of necessary risks involved in the procedure Educate patients on correct methods of maintaining oral hygiene Identify infection and potential infection sites and provide treatment Prescribe antibiotics if deemed necessary Provide chlorhexidine or other antimicrobial mouthwash prior to the procedure	Perform high-risk operations prior to bisphosphonate therapy (if possible) Possible drug holiday can be considered
Post-procedure		
Maintaining good oral health (e.g., brushing teeth, flossing) Maintaining good general health (e.g., smoking cessation, controlling diabetes) Monitor symptoms	Provide chlorhexidine or other antimicrobial mouthwash following procedure Prescribe antibiotics if deemed necessary Educate patients on symptoms to look out for	Follow-up appointments should be scheduled to monitor for symptoms of BRONJ

## Data Availability

The data presented in this study are available in this article.
